# Our First Competition

**Published:** 1914-05-09

**Authors:** 


### THE PRACTICAL EQUIPMENT OF A HOSPITAL.

### SUGGESTIONS FOR AN IDEAL WARD LOCKER.

### Our First Competition.

In accordance with the terms of the Prize Scheme announced in our issues of March 21 and 28 and April 11, we now publish the result of the judges' decision of the numerous papers which have been sent in in connection with this competition. The six illustrated descriptions which follow are considered to illustrate the most desirable features of a ward locker; but, in accordance with the terms of the competition, it is left to our readers to decide from their personal experience which of the lockers described most nearly approaches the ideal. This we ask them to do by means of votes, to be recorded on the voting paper on page 164. The competitor whose description secures the greatest number of votes will receive the first prize of five guineas, whilst the second prize of two guineas will be awarded to the competitor whose description secures the second highest number of votes. To that reader whose voting paper is found to agree most nearly with the majority of opinions expressed by the votes a special prize of three guineas will be awarded. Directions for voting will be found with the voting paper at the end of this article.

